# Childhood epilepsy and ADHD comorbidity in an Indian tertiary medical center outpatient population

**DOI:** 10.1038/s41598-018-20676-8

**Published:** 2018-02-08

**Authors:** Anita Choudhary, Sheffali Gulati, Rajesh Sagar, Naveen Sankhyan, Kam Sripada

**Affiliations:** 1 Department of Pediatrics, All India Institute of Medical Sciences, New Delhi, India; 2 Department of Pediatrics, Sawai Man Singh Medical College, Jaipur, India; 3 Department of Psychiatry, All India Institute of Medical Sciences, New Delhi, India; 4 Department of Pediatrics, Post Graduate Institute of Medical Education and Research, Chandigarh, India; 5 Department of Clinical & Molecular Medicine, Faculty of Medicine & Health Sciences, Norwegian University of Science & Technology, Trondheim, Norway

## Abstract

This study aimed to assess the prevalence of Attention Deficit Hyperactivity Disorder (ADHD) and its characteristics and risk factors in children with epilepsy at a tertiary medical center in New Delhi. Children with active epilepsy, aged 6 to 12 years, were assessed for ADHD using DSM-IV-TR criteria. Epilepsy and psychiatric characteristics, sociodemographic indicators, and use of antiepileptic drugs were analyzed for differences between the ADHD and non-ADHD groups. Among the 73 children with epilepsy, 23% (n = 17) had comorbid ADHD, of whom 59% (n = 10) had predominantly inattentive type, 35% (n = 6) combined type, and 6% (n = 1) predominantly hyperactive-impulsive type. Lower IQ scores, epileptiform EEG activity, not attending school, and male sex were significantly associated with comorbid ADHD in children with epilepsy. Groups were similar in terms of age, socioeconomic indicators, family history of psychiatric disorders, seizure frequency in the last six months, seizure etiology, and seizure type. Epilepsy is a common pediatric neurological condition with frequent psychiatric comorbidities, including ADHD. Specialists should collaborate to optimize treatment for children with epilepsy and ADHD, especially for families in developing countries where the burden of disease can be great.

## Introduction

Attention Deficit Hyperactivity Disorder (ADHD) is a common comorbidity experienced by children with epilepsy, and is associated with problems in social functioning, learning, quality of life, and academic attainment^1–4^. Several factors may contribute to this comorbidity^5,6^, including underlying brain pathology, chronic effects of seizures, epileptiform brain activity as seen on electroencephalogram (EEG), and effects of antiepileptic drugs^7^ (AEDs). This comorbidity also presents challenges to clinicians in determining a medication regimen and treatment plan^8^.

The reported prevalence of ADHD in children with epilepsy from high-income countries ranges from 23 to 70%, depending on sample studied and criteria used for diagnosis^1,9–11^, which is much higher than the reported worldwide pooled prevalence of ADHD of 5.3%^12^. Prevalence estimates of behavioral comorbidities in epileptic children and adolescents in India ranges from 40 to 54%^4,13,14^. In India, several studies have investigated psychopathology in children with epilepsy^4,13–18^; however, research focused on characterizing the ADHD comorbidity in epilepsy has been limited^19^.

This study aimed to quantify the prevalence of ADHD and its subtypes among children with epilepsy at an urban tertiary medical center. Measures of epilepsy severity, academic and sociodemographic factors, and use of AEDs were compared to describe the clinical profile of this pediatric group.

## Methods

### Participants

In this cross-sectional, observational study, children diagnosed with active epilepsy attending the outpatient services of the Child Neurology Division, Department of Pediatrics, All India Institute of Medical Sciences (AIIMS), New Delhi, were screened for eligibility. Active epilepsy was defined as two or more unprovoked seizures occurring 24 hours apart after four weeks of age, with at least one epileptic seizure in the previous five years, regardless of AED treatment, based on International League Against Epilepsy definitions^20^. The inclusion criteria were (1) children aged 6 to 12 years and (2) duration of epilepsy ≥ 6 months. Patients were excluded if they had an intellectual disability or comorbid chronic systemic disease. In total, 512 patients were screened for eligibility, 172 fulfilled the inclusion criteria, 62 had IQ ≤ 70, and 10 had comorbid systemic illness. Thus 100 patients were enrolled in a general epilepsy and psychopathology cohort (ages 6 to 16) whose findings have been previously reported^13^, and those aged 6 to 12 (n = 73) were included in this ADHD study.

Patients’ health records were retrospectively reviewed during the clinical course of their epilepsy. Epilepsy-related factors included age at seizure onset, duration of epilepsy, etiology, predominant seizure type, EEG pattern, seizure frequency in last six months, and AED therapy. Socioeconomic status categorization was based on monthly income per Agarwal’s six-stage classification^21^. Data collection took place between October 2008 and August 2009.

### Cognitive assessment

IQ was evaluated with Malin’s Intelligence Scale for Indian Children^22^ (MISIC), an Indian adaptation of the Wechsler Intelligence Scale for Children. MISIC assesses verbal IQ, performance IQ, and full-scale IQ for children between 6 and 16 years of age. For the present study, verbal IQ was assessed, encompassing six domains: information, general comprehension, arithmetic, analogies and similarities, vocabulary, and digit span. Intellectual disability was defined as a verbal IQ of 70 or lower.

### Psychiatric evaluation

Preliminary assessment of behavior problems used parent/caregiver ratings on the Child Behavior Checklist^23^ (CBCL/4–16). For participants scoring above the CBCL total score threshold for their age and sex^24^, an experienced psychiatrist and pediatric neurologist used the Diagnostic and Statistical Manual of Mental Disorders, 4th Edition, Text Revision^25^ (DSM-IV-TR) criteria for evaluation and diagnosis of 15 clinical disorders, including ADHD. Seventeen participants met the diagnostic criteria for ADHD.

### EEG assessment

Interictal digital EEG recording (Medelec profile; Oxford Instruments, Oxfordshire, UK) using an international 10–20 system of electrode placement was done at AIIMS for participants who did not have a good-quality EEG recording from the previous three months. Both awake and sleep states were recorded. Hyperventilation, photic stimulation, and sleep were used as activation procedures. EEG records were reported by two pediatric neurologists as either normal or showing epileptiform discharges.

### Statistics

IBM SPSS version 24 (Chicago, USA) was used for univariate analyses and descriptive statistics. Independent samples *t*-tests were used for normally distributed continuous variables. Chi-square (χ^2^) tests for association were used for categorical variables. The Mann-Whitney U Test was used for epilepsy duration and IQ, which had nonparametric distributions as assessed by Shapiro-Wilk’s test (*p* < 0.05). Post-hoc pairwise comparisons using Fisher’s exact tests (2 × 2) were used for seizure frequency.

### Ethics

This study was approved by the Institutional Ethics Committee at AIIMS, and the study was carried out in accordance with the relevant guidelines and regulations. Informed consent was obtained from the parents/caregivers of all participants.

### Data availability

Data analyzed during this study are included in the Supplementary Dataset.

## Results

### ADHD and sociodemographic characteristics

Of the 73 participants, 17 (23.3%) had both epilepsy and ADHD: within this group, 10 (58.8%) had ADHD predominantly inattentive type, 6 (35.3%) had ADHD combined type, and one (5.9%) had ADHD predominantly hyperactive-impulsive type.

Sociodemographic and family background information is presented in Table 1. IQ was significantly lower by almost 1.5 standard deviations in the ADHD group compared to participants without ADHD (mean score 84 vs 92, *p* < 0.0001). The ADHD group had significantly more males. Almost a quarter of the ADHD group did not attend school compared to 1.8% of non-ADHD participants (χ^2^(1) = 9.664, *p* = 0.0019), with epilepsy given in all cases as a reason for not attending. The group difference in school attendance was not driven by age. Seven participants with ADHD (41.2%) met the diagnostic criteria for an additional behavioral disorder, while 14 participants (25%) in the non-ADHD group did (see Supplementary Dataset). Age, socioeconomic indicators, and family history of psychiatric disorders were similar between groups.

**Table 1:** Sociodemographic and family profile of participants with and without ADHD, presented with group differences.

	Epilepsy with ADHD (n = 17)		Epilepsy without ADHD (n = 56)			
	Mean (SD)	Range	Mean (SD)	Range	χ^2^	p-value
**Age**, months	102.2 (20.1)	72–134	103.5 (22.1)	72–138	—	0.382
**IQ**	83.9 (8.4)	71–100	92.3 (6)	80–106	—	<0.0001
	**N (%)**		**N (%)**		**χ** ^**2**^	***p***-**value**
**Sex**						
Male	15 (88.2%)		33 (58.9%)		4.974	0.026
Female	2 (11.8%)		23 (41.1%)			
**Monthly income, Indian rupees**						
>10000	1 (5.9%)		1 (1.8%)		2.086	0.84
5000–9999	1 (5.9%)		8 (14.3%)			
3000–4999	1 (5.9%)		4 (7.1%)			
1500–2999	5 (29.4%)		13 (23.2%)			
500–1499	9 (52.9%)		29 (51.8%)			
<500	0		1 (1.8%)			
**Maternal education**						
<5th	4 (23.5%)		13 (23.2%)		0.858	0.84
Primary	7 (41.2%)		17 (30.4%)			
Secondary	3 (17.6%)		12 (21.4%)			
Graduation	3 (17.6%)		14 (25%)			
Single parent	0		2 (3.6%)		0.624	0.43
Family history of psychiatric disorders	1 (5.9%)		2 (3.6%)		0.177	0.67
**School attendance**						
Attend	13 (76.5%)		55 (98.2%)		9.664	0.0019
Not attend	4 (23.5%)		1 (1.8%)			

Chi-square values provided for categorical data. Abbreviations: ADHD: attention-deficit hyperactivity disorder; SD: standard deviation.

### Epilepsy characteristics

Comparison of clinical epilepsy characteristics is presented in Table 2. Age at onset of epilepsy ranged from one month to 11 years of age. Epilepsy duration ranged from 6 months to 10 years 5 months. Epileptiform discharges on EEG were positively associated with ADHD diagnosis (χ^2^(1) = 4.718, *p* = 0.030), with 52.9% of children with both epilepsy and ADHD showing epileptiform discharges, compared with 25% of those without ADHD. The ADHD and non-ADHD groups were comparable in terms of seizure etiology and seizure type. Post-hoc analysis of seizure frequency in the last six months did not indicate group differences in any of the frequency categories.

**Table 2:** Association of ADHD status and clinical epilepsy characteristics.

	Epilepsy with ADHD (n = 17)		Epilepsy without ADHD (n = 56)			
	Mean (SD)	Range	Mean (SD)	Range	χ^2^	*p-*value
**Epilepsy duration**, months	44.4 (26.8)	10–125	37.7 (27.4)	6–115	—	0.308
**Age at onset**, months	58.6 (29.4)	1–102	70.2 (27.7)	4–132	—	0.712
	**N (%)**		**N (%)**		**χ** ^**2**^	***p-*** **value**
**Etiology**						
Idiopathic	11 (64.7%)		43 (76.8%)		0.026	0.32
Symptomatic	6 (35.3%)		13 (23.2%)			
**Seizure type**						
Generalized	8 (47.1%)		30 (53.6%)		0.222	0.64
Partial	9 (52.9%)		26 (46.4%)			
**EEG**						
Normal	8 (47.1%)		42 (75%)		4.718	0.030
Epileptiform discharges	9 (52.9%)		14 (25%)			
**Seizure frequency in last 6 months**						
No seizures	6 (35.3%)		24 (42.9%)		3.589	0.17
1–10 seizures	7 (41.2%)		28 (50%)			
>10 seizures	4 (23.5%)		4 (7.1%)			
**Use of AED polytherapy**						
No AED	0 (0%)		3 (5.4%)		2.952	0.086
Monotherapy	11 (64.7%)		44 (78.6%)			
Polytherapy	6 (35.3%)		9 (16.1%)			

Chi-square values provided for categorical data. Abbreviations: ADHD: attention-deficit hyperactivity disorder; AED: antiepileptic drug; EEG: electroencephalogram; SD: standard deviation.

The following AEDs were used by study participants: valproate (27 participants), phenytoin (22), carbamazepine (20), clobazam (9), lamotrigine (6), oxcarbazepine (3), clonazepam (1), levetiracetam (1), and zonisamide (1). Three participants reported not currently taking any AEDs. Use of AED polytherapy was positively associated with ADHD diagnosis, but not significantly (χ^2^(1) = 2.952, *p* = 0.086).

## Discussion

At a tertiary medical care center in New Delhi, a high prevalence (23.3%) of ADHD was identified in children with epilepsy. In agreement with several previous studies^9,12^, ADHD inattentive type was more frequent in this clinical group than hyperactive-impulsive or combined types. Most epilepsy characteristics did not differ significantly between the groups with or without ADHD, but abnormal EEG was associated with ADHD diagnosis. Children with both epilepsy and ADHD had lower IQ scores and were significantly less likely to be attending school, with epilepsy being the primary reason. Sociodemographic profiles of the two groups were similar.

This prevalence rate is similar to the 23.4% of children with epilepsy and comorbid ADHD reported by Philip *et al*.^19^ in a pediatric neurology outpatient population in Karnataka, India (n = 94). A recent register-based study in Sweden^26^ of 1,899,654 individuals found that 13.5% of those with epilepsy also had a diagnosis of ADHD, while 4.3% of the full sample had ADHD, meaning a 3.5-fold increased risk in the comorbid group. In Asia, recent reports from Thailand^27^ and China^28^ found prevalences of 19% and 42%, respectively, of ADHD comorbidity among children with epilepsy. Studies of ADHD prevalence in India are limited, use different methodologies, and report varying figures. Recently reported prevalence figures of ADHD in children in the general population include 1.3% in a study of children in Bengaluru, Karnataka^29^ (n = 3120), 1.7% in a study of families in central India^30^ (n = 4278 families), 11.32% in a study of children in Coimbatore, Tamil Nadu^31^ (n = 770), and 12.66% in a study of children in the state of Assam^32^ (n = 300).

The population of children with comorbid epilepsy and ADHD differs in several ways from those with primary ADHD. For instance, the gender distribution is less skewed towards boys than in primary ADHD, and inattention symptoms are more frequent and severe than impulsivity/hyperactivity^33,34^, in line with the results presented here. In an American study, Dunn *et al*.^35^ found that of children aged 9 to 14 with epilepsy, 24% had ADHD inattentive type, compared to 14% with hyperactive-impulsive or combined types. In a Nigerian tertiary care facility, Chidi *et al*.^36^ found that of the 14% of epileptic children who also had ADHD, the inattentive type was predominant (69%).

### Biological mechanisms

Recent neuroimaging research using magnetic resonance imaging (MRI) has sought to shed light on possible shared neural mechanisms underlying epilepsy and ADHD comorbidity^1,10,37–39^. While some evidence implicates shared neurobiological abnormalities^40,41^, other studies suggest that independent factors are at play^10,40^. Regions of decreased cortical thickness have been reported in groups with comorbid epilepsy and ADHD, compared to epilepsy alone, which could indicate altered development of functional networks^42^. Saute *et al*.^38^ reported that pediatric epilepsy with comorbid ADHD was associated with reduced cortical thickness in bilateral areas of the frontal, parietal, and temporal regions, along with smaller caudate, thalamus, hippocampus, and brainstem volumes, compared to the epilepsy non-ADHD group; smaller cerebellum and thalamus were related to epilepsy generally, but brain volume and surface area did not show major group differences. Similarly, Dabbs *et al*.^43^ reported that ADHD problems assessed with the CBCL were associated with decreased cortical thickness in left superior frontal and lateral occipital regions and right fusiform gyrus. In a functional MRI study of boys with ADHD, Bechtel *et al*.^44^ reported similar patterns of brain activity during block-design working memory tasks in the groups with and without comorbid epilepsy, which differed from healthy controls. Neural mechanisms underlying epilepsy and ADHD, as opposed to ADHD or epilepsy alone, remain to be fully elucidated and will benefit from further neuroimaging research.

Genetic studies, especially at the population level, have also begun to identify risk factors related to these and other neurodevelopmental disorders^45^. Relatives of those with epilepsy have an increased risk of ADHD themselves^26^, suggesting genetic and environmental linkages within families, and evidence of an association between maternal epilepsy and ADHD has also been reported^46^. However, Brikell *et al*.^26^ found only a modest genetic correlation between the disorders. In addition to inherited genetic factors, *de novo* mutations and other rare variants, such as those involved in synaptic function, neurotransmission, and methylation remodeling, should be investigated as potential risk factors (see review by Lo-Castro and Curatolo^47^).

### Treatment challenges and AEDs

A clearer understanding of medication effects on the symptomatology and progression of combined epilepsy and ADHD is needed to tailor medical management in this population^34^. It is possible that epilepsy alters the ADHD symptoms in children who have both, based on possible shared etiology and pathophysiology^44,48^. For example, children with ADHD are predisposed to epileptic seizures^40^ and have an increased rate of EEG abnormalities without a history of epilepsy^49^.

Determining an effective treatment regimen for children with epilepsy and ADHD is challenging, as clinicians must consider a patient’s seizure frequency, cognitive function, drug interactions, and medication adherence. Use of AEDs, in particular polytherapy, has in some studies been associated with cognitive deficits in children including attention problems and hyperactivity^50–52^. Sleep disturbances due to AEDs, along with subclinical seizures and learning disabilities, can contribute to inattention^51^, which was the ADHD subtype diagnosed in 59% of children with ADHD in this study. Several studies suggest that use of methylphenidate appears to be effective in children with comorbid epilepsy and ADHD^49,52,53^. A 2015 Brazilian study^54^, which administered low to moderate doses of methylphenidate, observed reduced seizure frequency and severity along with improved quality of life among adolescents with ADHD and difficult-to-treat epilepsy. However, increased risk of seizures with high dosage of osmotic release oral system methylphenidate^55^ and lowered seizure threshold associated with tricyclic antidepressants and bupropion use has also been reported^1,41,52,56^. Most studies of medication for ADHD in children with epilepsy have had small numbers of participants and/or short study duration^52^, and clinical trials in developing countries have been limited^57,58^. Research on other medications, such as amphetamines, atomoxetine, and alpha-2 adrenergic agonists, has also been limited in this population. Medication effects on attentive behavior and seizure threshold should therefore be closely monitored. EEG monitoring is a useful tool in this population to identify whether staring episodes or repetitive movements are related to seizure activity or ADHD. Children with epilepsy in this study experienced ADHD at a high rate, and further research in larger populations should therefore clarify the risk-benefit ratios associated with various medications.

Moreover, Gonzalez-Heydrich *et al*.^59^ report that children with epilepsy and ADHD often have additional comorbidities, such as anxiety or oppositional defiant disorder, making treatment decisions complex^33^. AED polytherapy may also create financial stress for some families^3^. Holistic management of children with epilepsy would include screening and management of psychiatric problems, including ADHD. School failure and drop-out was a problem for a number of children in this study and underscores the need to engage the education community in this region to improve academic outcomes^60^. Further studies are required to evaluate the efficacy of behavior therapy, drug therapy, and other treatment modalities for individuals with epilepsy and ADHD, as well as their effect on improving quality of life^61^.

### Strengths and limitations

Limited research in India has examined ADHD comorbidity in children with epilepsy using diagnostic criteria. In this study, standard criteria from the DSM-IV-TR were used for diagnosing ADHD. Participants were recruited from the outpatient population of a government hospital which serves children from all socioeconomic backgrounds.

The study population was drawn from the tertiary care center outpatient population, and all participants in this study had a history of epilepsy, making it difficult to generalize to primary care settings and to the typically developing pediatric population. Future studies should therefore also include a healthy matched group without epilepsy. Within-group analysis, for example more detailed analysis of seizure frequency related to comorbidity, would be strengthened by a larger number of participants. While Malin’s Indian adaptation of the WISC has been widely used across India for cognitive testing in children, future studies should consider using newer assessments, such as the WISC-IV India. Finally, the revised DSM-V was released after the data collection in this study. Using the updated diagnostic criteria for ADHD may have classified additional children with ADHD, meaning that the prevalence figures reported here are likely conservative. The DSM-V designation of mild, moderate, or severe ADHD would have provided extra detail to the current study.

## Conclusion

Prevalence of comorbid ADHD was 23% in children with epilepsy attending outpatient services at a tertiary medical center in New Delhi, with inattentive type being predominant. In this study, children with comorbid ADHD had lower IQ scores on average, and four of the five children who did not attend school had both ADHD and epilepsy. Clinicians should be sensitive to identify ADHD in children with epilepsy since this may lead to more effective intervention and improved quality of life^62^. Further research examining potential shared neurological causes as well as strategies to optimize medication and treatment are needed to better serve this pediatric population.

## Electronic supplementary material

Supplementary Information
